# Cold water swimming as an add-on treatment for depression. A feasibility study

**DOI:** 10.1192/j.eurpsy.2022.1432

**Published:** 2022-09-01

**Authors:** P. Hjorth, A. Løkke, N. Jørgensen, A. Jørgensen, M. Rasmussen, M. Sikjaer

**Affiliations:** 1 Region of Southern Denmark, Department Of Psychiatry Vejle, Vejle, Denmark; 2 Region of Southern Denmark, Department Of Intern Medicine, Vejle, Denmark; 3 Region of Southern Denmark, Internal Medicine, Vejle, Denmark

**Keywords:** Depression, prevention, cold water swimming

## Abstract

**Introduction:**

In Denmark, about 14% of patients with depression develops treatment resistant depression (TRD) in the following year after the first hospital contact. Possible explanations for TRD include lack of adequate clinical effect of pharmacological treatment and reluctance to treatment due to unacceptable side effects. Cold water swimming (CWS), also known as winter swimming, describes swimming outdoors - mainly during the winter season in cold to ice-cold water on a regular basis. Many winter swimmers believe that exposure to cold water is beneficial for their health. However, evidence of health effects have been anecdotal or based on results from small sample-size studies. The availably studies report that winter swimming abolishes general tiredness, boosts self-esteem and improves mood and/or general well-being.

**Objectives:**

To test if it is possible for patients with depression to participate in two weekly sessions of CWS and to measure the effects of CWS on general well-being and depression among the participating patients.

**Methods:**

All psychiatric in- and outpatients from the department of psychiatry at Little Belt Hospital, Vejle with a diagnose of depression are eligible for inclusion. CWS-sessions will include a dip in an inlet - and if desired a short swim for a few minutes – depending on individual preferences. The CWS sessions will take place at the local inlet at a recreational area with sauna and changing facilities available.

**Results:**

The study starts in October 2021 and we expect to have results by April 2022.

**Conclusions:**

Conclusion: Awaiting.

**Disclosure:**

No significant relationships.

